# The hippocampus is required for visually cued contextual response selection, but not for visual discrimination of contexts

**DOI:** 10.3389/fnbeh.2012.00066

**Published:** 2012-09-28

**Authors:** Sehee Kim, Jihyun Lee, Inah Lee

**Affiliations:** Department of Brain and Cognitive Sciences, Seoul National UniversitySeoul, Korea

**Keywords:** hippocampus, context, navigation, episodic memory, response selection, choice behavior, decision making

## Abstract

The hippocampus is important for spatial navigation. Literature shows that allocentric visual contexts in the animal's background are critical for making conditional response selections during navigations. In a traditional maze task, however, it is difficult to identify exactly which subsets of visual contexts are critically used. In the current study, we tested in rats whether making conditional response selections required the hippocampus when using computer-generated visual contextual stimuli in the animal's background as in primate and human studies. We designed a new task, visual contextual response selection (VCRS) task, in which the rat ran along a linear track and encountered a touchscreen monitor at the end of the track. The rat was required to touch one of the adjacent rectangular box images depending on the visual contextual stimuli displayed in the two peripheral monitors positioned on both sides of the center touchscreen monitor. The rats with a GABA-A receptor agonist, muscimol (MUS), infused bilaterally in the dorsal hippocampi showed severe performance deficits in the VCRS task and the impairment was completely reversible with vehicle injections. The impairment in contextual response selection with hippocampal inactivations occurred regardless of whether the visual context was presented in the side monitors or in the center touchscreen monitor. However, when the same visual contextual stimuli were pitted against each other between the two side monitors and as the rats simply ran toward the visual context associated with reward on a T-shaped track, hippocampal inactivations with MUS showed minimal disruptions, if any, in performance. Our results suggest that the hippocampus is critically involved in conditional response selection using visual stimuli in the background, but it is not required for the perceptual discrimination of those stimuli.

## Introduction

What is learned in a certain environment is best remembered in the same environment. This so-called contextual effect on memory retrieval has long been studied in both humans and animals (Hirsh, 1974; Godden and Baddeley, 1975; Smith, 2001; Fanselow, 2007). The hippocampus is important for such contextual behavior, which includes making discrete choice responses depending on what is present in the animal's background (Hirsh, 1974). In the literature, the stimuli in the animal's background that provide critical conditional information for response selection are collectively and customarily called a “context” or contextual cue (Winocur and Olds, 1978; Good and Honey, 1991; Kim and Fanselow, 1992; Yeshenko et al., 2004; Lee and Solivan, 2008; Lee and Shin, 2012). In humans and primates, visual stimuli have been predominantly used as contextual cues for testing hippocampal functions in conditional choice making (Gaffan and Parker, 1996; Murray et al., 1998; Burgess et al., 2002; King et al., 2002; Ekstrom et al., 2003; Wirth et al., 2003; Hampson et al., 2004; Howard et al., 2011). For example, hippocampal neurons showed learning-related physiological correlates when monkeys were required to make a saccadic eye movement in a particular direction in association with a visual scene presented in a computer monitor (Wirth et al., 2003). In humans, functional imaging studies that involved navigation in a virtual environment also required the subjects to make a series of response selections in association with the visual stimuli (e.g., scenes) presented in a computer monitor (Burgess et al., 2001; Ekstrom et al., 2003; Hartley et al., 2003).

In rodent studies, the term “context” has been used in a broader sense to include spatial, visual, temporal, and even internal variables, to name a few (Hirsh, 1974; Winocur and Olds, 1978; Kesner and Hardy, 1983; Wible et al., 1986; Kim and Fanselow, 1992; Hock and Bunsey, 1998; Kennedy and Shapiro, 2004; Vazdarjanova and Guzowski, 2004; Smith et al., 2012). In a typical contextual fear conditioning experiment (Kim and Fanselow, 1992), for example, a context is defined as a mixture of unspecified room cues, ambient odors in a conditioning chamber, and other cues (e.g., housing light, fan sound, and other elemental cues inside the chamber, etc.). Multimodal sensory inputs (mainly via the entorhinal cortex) and other subcortical inputs into the hippocampus may justify such an inclusive definition of the term context (Witter and Amaral, 2004). However, in order to establish firm functional relationships between a neural cognitive process and behavior, it is essential to use well-defined controlled stimuli to know exactly what the inputs to the system under investigation are. Because of the comprehensive definition of context in rodent studies as described above, despite the well-established roles of the hippocampus in contextual learning and memory using such stimuli, several caveats are identified while investigating the neural mechanisms. First, it is difficult to specify *a priori* the relative contributions of different subsets of sensory cues in the environment. This problem becomes particularly salient when a context is operationally defined as an unspecified mixture of sensory cues in the environment (e.g., entire room). In typical spatial memory tasks using, for example, the Morris water maze (Morris et al., 1982) or 8-arm radial maze (Olton and Samuelson, 1976), it is difficult to specify exactly which cues “out there” are associated with particular behavioral selections made during spatial navigation despite the well-known dependence of those tasks on allocentric visual cues. Second, the traditional contextual manipulations (e.g., switching between different rooms) often make it difficult to test how hippocampal networks process contextual cues *dynamically* because it is practically implausible to switch between different contexts on a trial-by-trial basis while maintaining all other cues exactly the same when real environmental cues (e.g., different rooms, cue cards, odors, etc.) are used. Finally, although it is well established that the hippocampus is particularly important when similar contexts need to be generalized or discriminated (Gilbert et al., 1998; Lee et al., 2004; Leutgeb et al., 2004; Vazdarjanova and Guzowski, 2004; Gold and Kesner, 2005), it is difficult to parametrically manipulate the level of ambiguity in the surrounding context along a single sensory dimension (e.g., vision) in the traditional contextual paradigm.

If rodent hippocampal functions can be tested effectively using well-defined stimuli as in primate and human visual contextual research fields, it will make a significant improvement in studying the role of the hippocampus by minimizing the confounding problems mentioned above that arise when using vaguely defined contextual stimuli. In the current study, we tested whether rats can perform contextual response selection using purely visual stimuli presented in the animal's background as similarly as in primates and humans. It has been previously shown that rats can visually discriminate 2D images of objects and visual patterns (Gaffan and Eacott, 1995; Prusky et al., 2004; Forwood et al., 2007; Bussey et al., 2008; Talpos et al., 2009) and also can perform virtual spatial navigations using such stimuli (Harvey et al., 2009; Dombeck et al., 2010). Among those studies, to our knowledge, only Prusky and colleagues tested the roles of the hippocampus by making hippocampal lesions (Prusky et al., 2004). In that study, however, patterned visual stimuli were presented as navigational “targets” toward which rats must swim in a water maze. Since the rats were only required to discriminate the two visual stimuli and swim toward the side on which the visual stimulus associated with a submerged platform was displayed, the task was more of a perceptual discrimination task than a contextually cued response-selection task. In the current study, the rats were required to choose one of the target locations in a touchscreen when cued by a visual context presented in the background. We report here that visual contextual cueing of response selection is critically dependent on the dorsal hippocampus, whereas perceptual discrimination of the visual contexts is relatively unaffected in the absence of the normal function of the hippocampus.

## Materials and methods

### Subjects

Eighteen rats (Long-Evans, male, 250–400 g) were used. Upon arrival, rats were housed individually in Plexiglas cages in a temperature and humidity-controlled environment. All animals were maintained on a 12 h light/dark cycle. Each rat was allowed to access to water *ad libitum* and food-deprived to 80% of its free-feeding weight for behavioral testing. Before handling, rats were given 2 weeks to acclimate to the environment. All protocols conformed to the NIH guide for the Care and Use of Laboratory Animals and the Institutional Animal Care and Use Committee of the Seoul National University.

### Behavioral apparatus

**Figure 1:**
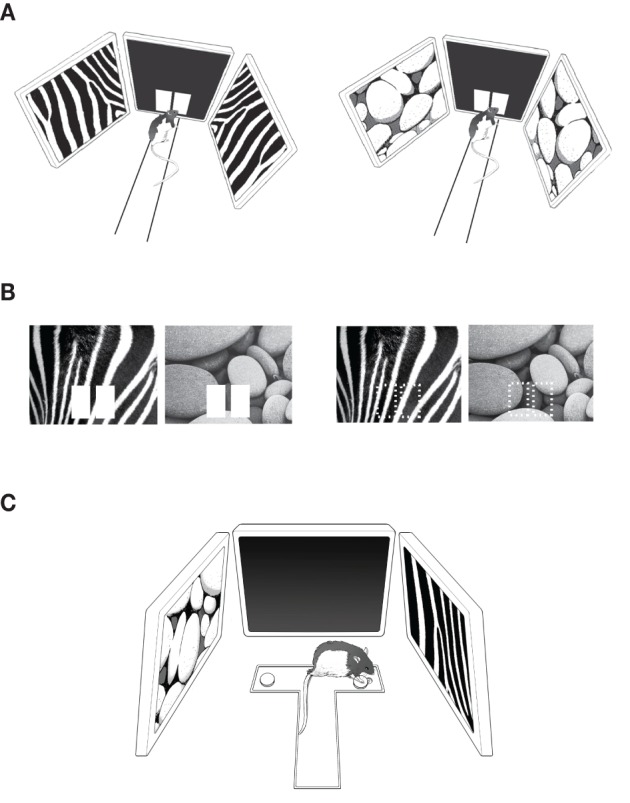
**Behavioral paradigms. (A)** The pVCRS task. The rat was required to touch one of the response boxes (white rectangular images) presented in the center touchscreen monitor by using peripheral visual contexts (zebra and pebbles patterns) in the side monitors. The start box was omitted for illustration purposes. **(B)** The cVCRS task. The visual context was presented in the center touchscreen as a background of the response boxes (*left*) or by itself without the response boxes (*right*; the response areas are indicated by dotted rectangles for illustration purposes only). Only the center touchscreens were illustrated here without peripheral monitors and the apparatus for simplicity. **(C)** The VPD task. The two visual contexts were presented via the side monitors and the rat chose the correct arm (right arm associated with the zebra pattern in this example) of the T-track after entering the center stem. Displacing the metal washer revealed a food well and the rat retrieved a food reward from the food well.

Rats were trained on an elevated linear track (43 × 8 cm; 84 cm above the floor) with a rectangular start box (15 × 22 × 39 cm) at one end and an array of LCD monitors (elevated 13 cm from the linear track surface) at the other end (Figure 1A). A 15-inch LCD monitor (AccuSync L154F0 TFT model, NEC; maximum viewing angle = 120° horizontally and 90° vertically) in the center and two 17-inch LCD monitors (Syncmaster MC17PS TFT LCD model, Samsung; maximum viewing angle = 160° horizontally and 90° vertically) on both sides were installed as an array with the midline of the center monitor aligned with the midline of the track. The angle made by the lateral edges of the center monitor and its adjacent peripheral monitors was 98°. The center monitor's bottom was tilted upward by 13° and each peripheral monitor's bottom was tilted downward by 10° for providing best viewing angles for visual stimuli to the rats. The center monitor was equipped with a touchscreen panel (Elo TouchSystems, Menlo Park, CA) to register a touch response using infrared beams. All monitor's refresh rates were set at 60 Hz. A transparent Plexiglas panel (35.4 × 27.3 × 0.15 cm) with two rectangular openings (each 6 × 10 cm), each serving as a response window, was attached to the touchscreen panel. During the task, two white rectangular images (“*response boxes*” hereafter, each 4.6 × 8.6 cm, RGB value 255-255-255; 0.2 cd/m^2^) were presented on black background (RGB value 30-30-30) in alignment with the response windows in the center monitor (Figure 1A) and the rats touched either response box to indicate their choices. A food tray was installed below the center monitor and the rats retrieved a ball-shaped cereal reward (Coco balls, Kellogg's) from the tray during the task. There were three optic fiber sensors (Autonics, Busan, Korea) installed along the track (at both ends and in the middle of the track). Breakage of each sensor beam was registered via a data acquisition board (SCB-68, National Instruments, TX) interfaced with a PC. The PC was equipped with a high-performance multi-view graphic card (Firepro MIV 2450, ATI). Custom-written software using Psychtoolbox (Brainard, 1997; Pelli, 1997) in Matlab (Mathworks, Natick, MA) controlled stimuli and registered touch responses as wells as sensor times. The apparatus was located in a sound-attenuating room and a digital CCD camera was positioned above the apparatus for recording behavioral sessions. A halogen light was installed immediately adjacent to the CCD camera for illuminating the room at 0.2 lux. Two loud speakers were placed in the behavioral testing room for providing white noise (80 dB) during behavioral testing.

### Handling, familiarization and shaping

Naïve rats (250 ~ 400 g) were handled approximately a week before shaping procedures began. On the first day of handling, rats were handled for 20 min to become acquainted to the experimenter. From the next day, the rats were handled for 10 min and freely foraged for cereal for 20 min to become familiarized with the food reward. That is, the rat was placed on a laboratory cart (outside the behavioral testing room) and several pieces of cereal were scattered randomly on the cart (78 × 44 × 83 cm). When the rat readily consumed multiple pieces of cereal, a shaping period began. During the shaping period, the rat was first familiarized to the apparatus and the testing room by letting the rat freely ate cereal pieces scattered along the track. Afterwards, the animal was trained to touch one of the response boxes on the touch screen panel to obtain a reward with a sound feedback (2 kHz, 3 s, 83 dB). When rats failed to touch a response box, an error sound (0.2 kHz, 3 s, 83 dB) was given and the animal was guided back to the start box without any reward. Throughout all tasks in the current study, rats voluntarily returned to the start box after making a choice on each trial. Each shaping period ended after either 50 trials or 30 min, whichever came first, and the whole shaping procedure lasted for 5 days.

### Behavioral pre-training

Fourteen rats were divided into two groups: Eight rats were assigned to a peripheral visual contextual response selection (pVCRS) task. The other six rats were used in a central VCRS (cVCRS) task and also in a visual pattern discrimination (VPD) task. A within-subjects design was used for drug injecting protocols throughout the study.

#### pVCRS task

Eight rats were pre-trained before surgery in a pVCRS task. In the task, the rat was required to touch one of the response boxes according to the patterned visual stimuli (“visual context” hereafter; visual patterns chosen from wallpaper images from the Apple desktop computer) presented in the peripheral LCD monitors (Figure 1A). Each trial started by presenting the mirrored images of the same visual context in the peripheral monitors and opening the start box door. One of the visual contexts (zebra pattern, Figure 1A *left*) was associated with the left response box and the other visual context (pebbles pattern, Figure 1A *right*) was associated with the right response box for reward. The context-response associations with reward contingencies were counterbalanced among the animals. Touching a correct response box resulted in an immediate sound feedback (2 kHz, 3 s, 83 dB; from the in-room experimental PC) followed by a piece of cereal reward in the food tray, whereas choosing a wrong response box produced an error sound (0.2 kHz, 3 s, 83 dB) without reward. An inter-trial interval of 4 s was given after a correct trial but a longer intertrial interval (15 s) was imposed after incorrect choices were made. Fifty trials were given within a session. Each visual context appeared an equal number of times within a session and the sequence of scenes was pseudorandomized. The rat was required to show ≥75% correct choices for both contexts to receive cannula implantation surgery. It took 5–12 days (median ≥ 7) for the rats to meet the surgical criterion. In addition to the percent correct score, the latencies to the sensors along the track were also measured.

#### cVCRS task

In the cVCRS task, rats (*n* = 10) were required to touch one of the response areas in association with the visual patterns presented on the center screen (Figure 1B). The response areas were marked by white rectangular images in most animals (*n* = 6; Figure 1B *left*). In some rats (*n* = 4), the response box images were not presented and the rats were required to touch the areas of the touch screen available through the rectangular cuts made in the acrylic panel on top of the touchscreen monitor (Figure 1B *right*). This was to test whether the rats perceived the response images as objects and whether performance changed depending on the availability of those response boxes. Unlike the pVCRS task, visual stimuli were not presented in the peripheral LCD monitors in the cVCRS task. The same visual contexts (i.e., zebra and pebbles patterns) that were used in the pVCRS task were presented in the center touchscreen monitor in a pseudo-random order. Each trial began by presenting one of the visual contexts on the center screen and then by opening the start box. Among the six rats that learned the version of the cVCRS task with response box images, three rats were assigned to touch the left response box when the zebra pattern appeared and the right response box for the pebbles pattern for obtaining reward. The other three rats followed the opposite stimulus-response contingency. A correct response resulted in an immediate sound feedback (2 kHz, 3 s, 83 dB) followed by a reward in the food tray, while a wrong response initiated an error sound (0.2 kHz, 3 s, 83 dB) with no reward. No correction was allowed once an incorrect response was made. An intertrial interval of 4 s was given after a correct trial but a longer intertrial interval (15 s) was imposed after an incorrect response before the next trial began. Fifty trials were given within a session. Each visual context appeared an equal number of times within a session and the presentation sequence of the contexts was pseudo-randomized. The rats that learned the cVCRS task without the response box images were trained equally with the only exception being that the response box images were unavailable. When the rats showed ≥75% correct performances for both visual stimuli for two consecutive days, they were considered ready for implantation surgeries for bilateral cannulae in the hippocampi. It took 11–20 days (mean ≥ 16.5) for the rats to reach performance criterion. In addition, the latency from the presentation of the visual context until the rat touched the touchscreen was measured via fiber optic sensors and the touchscreen.

### Surgery

Each rat was deeply anesthetized with isoflurane (4% mix with oxygen at a flow rate of 1 L/m) in an induction chamber, followed by an injection of Nembutal (70 mg/kg). The animal was placed in a stereotaxic instrument. The anesthesia was maintained by isoflurane (1–3%) throughout surgery. The skull was exposed and adjusted to place bregma and lambda on the same horizontal plane. After small burr holes were drilled, two sets of 26 G guide cannulae (Plastics One, Roanoke, VA) were implanted bilaterally into the dorsal hippocampus (3.9 mm anterior to bregma, 2.6 mm lateral to midline, 3.0 mm ventral from the skull surface). The cannulae were secured in place with anchoring screws and dental cement. A 32 G dummy cannula was inserted into each guide cannula to prevent clogging. Rats were allowed to recover for 7 days.

### Intracranial microinjection

After backfilling a 10 μL syringe (Hamilton, Reno, NY) with mineral oil, 33 G injection cannula was connected via polyethylene tubing (PE-20; Becton Dickinson). Either muscimol (MUS) or saline (SAL) was injected at the rate of 10 μL/h using a micro-infusion pump (KD Scientific, Holliston, MA). For injection, after dummy cannulae were moved, an injection cannula extending 1 mm below the tip of the guide cannula was inserted. Sterile SAL was used for the control conditions. A GABA-A receptor agonist, MUS (0.5 μg/0.5 μL), was bilaterally injected to temporally inactivate the dorsal hippocampus. The injection cannulae were left in place for an additional 1 min to ensure a proper diffusion of the drug. The rat was returned to its home cage and was examined for any abnormal movement for 20 min before the actual task began.

### Post-surgical behavioral testing paradigm

#### Testing performance in the pVCRS task

Post-surgical testing was conducted in the pVCRS task with or without the injection of MUS on the dorsal hippocampus as follows. After a week of recovery, the rats (*n* = 8) were retrained to criterion (≥75% correct choices for both scenes) in the pVCRS task without any drug infusion. Once the rat was trained to criterion, either SAL or MUS was injected into the hippocampus on the next day for four consecutive days with the injection schedule of SAL-MUS-MUS-SAL. MUS was injected for 2 days in a row in order to test whether learning occurred in the absence of normal hippocampal functions in the task.

#### Testing performance in the cVCRS task

After a week of recovery period, the rats (*n* = 10) that had been trained in the cVCRS task were retrained to criterion (≥75% correct performance for both scenes) in the same task. Then, the drug injection schedule of SAL-MUS-MUS-SAL began with a within-subject design for four consecutive days.

#### Training and testing performance in visual pattern discrimination (VPD) task

After the rats (*n* = 6) that were tested in the response box-version of the cVCRS task finished post-surgical testing, the same rats were trained in a VPD task to discriminate between the two visual contextual patterns used in the pVCRS and cVCRS tasks. The VPD task was conducted on a T-shaped track (35 × 35 × 7 cm) made of black Plexiglas (Figure 1C). The T-track was placed in the middle of the LCD-monitor array of the same behavioral apparatus used in the VCRS tasks. A food well (2.5 cm in diameter and 0.5 cm in depth) was located at the end of each arm of the T-maze and the food wells were always covered with identical small metal discs (each 3.5 cm in diameter). The food well of each arm of the T-maze was positioned in front of the peripheral LCD monitors (distance between the food well and the peripheral screen ≥ 16.5 cm) and the rat could retrieve a piece of cereal by displacing the disc over the food well (Figure 1C). On the first day of acquisition, the food wells were intentionally left half-open to show the food reward to the rats and the animals quickly learned to displace the overlying disc to retrieve the rewards. On the second day, the rat was forced to turn to a certain arm on each trial because access to a randomly chosen arm was blocked by a heavy plastic block (5 × 5 × 7 cm). From the third day onwards, rats were trained in the VPD task. Specifically, the visual patterns were presented in both peripheral monitors (i.e., the zebra pattern for one of the monitors and the pebbles pattern for the other monitor). The task for the rat was, once released from the start box and entering the stem of the T-maze, to simply enter the arm associated with the peripheral monitor showing the visual pattern that was associated with reward (Figure 1C). The center monitor was not used and remained in dark gray (RGB value 30-30-30). In a given behavioral session (50 trials), each visual pattern appeared an equal number of times with equal probabilities of appearance in both monitors. The rewarding visual pattern was counterbalanced among rats. Latency was measured from the start box to the moment the rat displaced the disc by a stopwatch. Once the rat acquired the task to criterion (≥75% correct performances for both arms over two consecutive days), the same drug injection schedule (SAL-MUS-MUS-SAL) started for four consecutive days.

### Histology

After the completion of all behavioral experiments, cannula positions were histologically verified. For this purpose, the rat was killed by the inhalation of a lethal dose of CO_2_ followed by a transcardial infusion of 0.9% SAL and a 4% formaldehyde solution. The brain was then extracted and stored in a 4% formalin-30% sucrose solution at 4°C for 48 h. The brain was frozen and cut in coronal sections (40 μm) on a sliding microtome (Thermo Fisher Scientific, Waltham, MA). The sections were then Nissl-stained with thionin (Sigma, St. Louis, MO), and examined under the light microscope. Only the rats with cannula tips located bilaterally within the dorsal hippocampi were included in the final data analysis.

## Results

### Histological verifications of cannula positions

**Figure 2:**
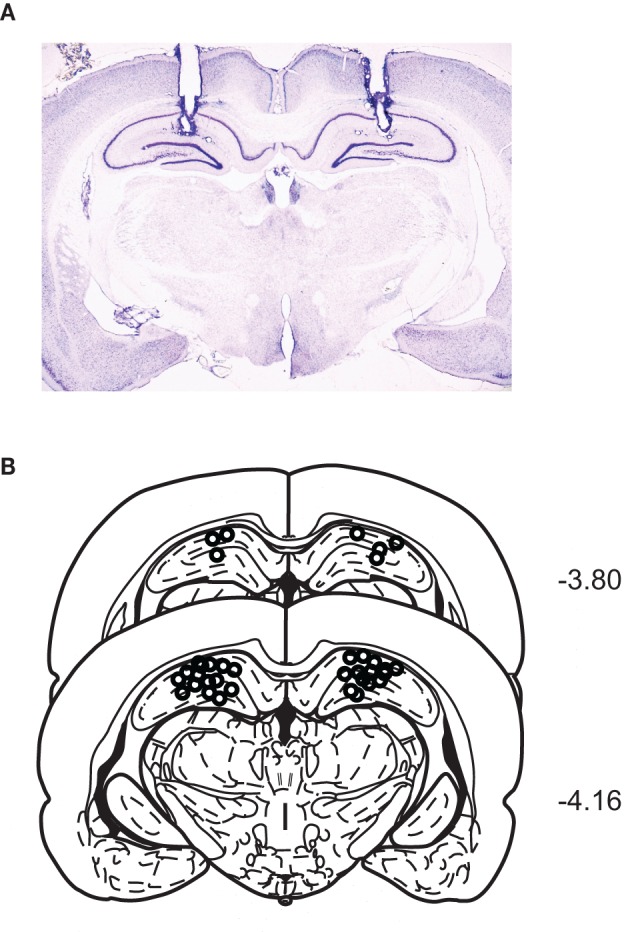
**Cannula positions in the dorsal hippocampus. (A)** A representative Nissl-stained section showing bilateral cannula tracks targeting the dorsal hippocampi. **(B)** Schematic illustration of the cannula-tip positions of all animals used in the current study.

A representative photomicrograph showing cannula tracks through the dorsal hippocampus is shown in Figure 2A. The cannula-tip positions of all rats were identified within the dorsal hippocampi (Figure 2B).

### The hippocampus is necessary in the pVCRS task

**Figure 3:**
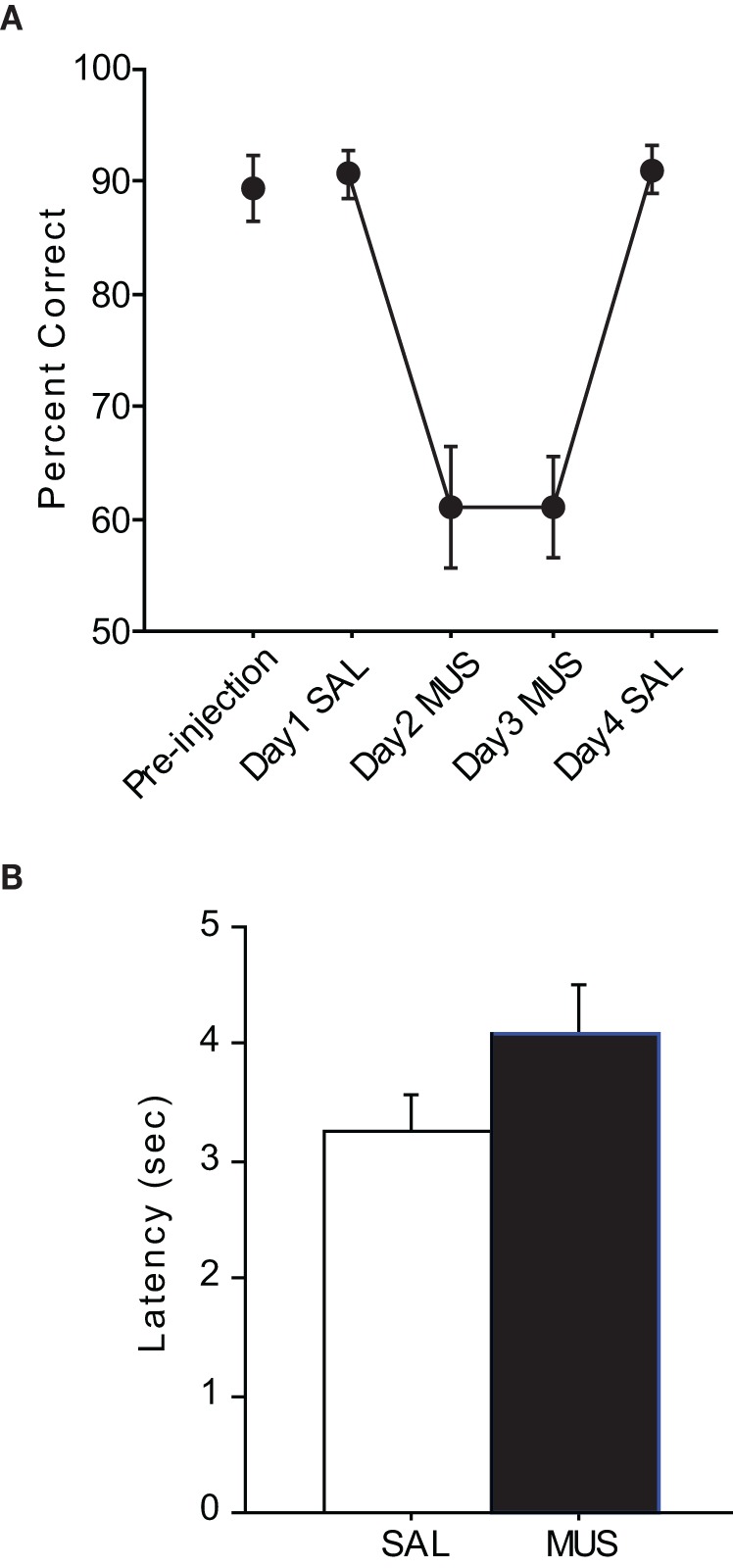
**Performance in the pVCRS task. (A)** Rats with MUS infusions in the hippocampi were severely impaired compared to the SAL conditions. **(B)** Average latency measures were not significantly different between SAL and MUS conditions. All graphs show mean ± s.e.m.

After the surgeries, all rats were re-trained to criterion before SAL was infused (Figure 3A). The performance with SAL was comparable to the pre-injection performance level [*t*_(5)_ = −0.38, *n*.*s*.]. However, MUS injections produced severe impairment in performance for the following two consecutive days and the MUS effects were completely reversed with SAL injections on Day 4. An ANOVA with repeated measures revealed a significant effect of drug-injection day [*F*_(5, 12)_ = 39.01, *p* < 0.001]. *Post-hoc* comparisons (Tukey-Kramer) demonstrated significant differences in performance between the SAL and MUS injections for the first 2 days (*p* < 0.001) and between the MUS and SAL injections during the last 2 days (*p* < 0.05). No significant difference was found between the Day-1 and Day-4 SAL injections or between the Day-2 and Day-3 MUS injections. Furthermore, there was no significant difference in performance for the two contextual conditions. Response latency from exiting the start box to touching a response box was measured to examine if MUS injections caused generic motor deficits that might have affected the performance (Figure 3B). There was no significant difference between the SAL and MUS conditions in response latency [*F*_(5, 15)_ = 0.73, *n.s*.]. Furthermore, paired *t*-tests showed no significant differences between the SAL and MUS conditions in traveling times between the sensors along the track. Overall, the results from the pVCRS task strongly demonstrate that using 2D visual contextual stimuli in the background for a simple conditional choice behavior imposes a significant cognitive demand and requires normal hippocampal functions.

### Inactivation of the dorsal hippocampus impairs performance in the cVCRS task

In the pVCRS task, the rats were required to respond to indicate their choices by touching the adjacent response boxes in the center touchscreen while viewing the visual contextual stimuli displayed in the peripheral monitors. It is possible that this distance between the peripheral visual stimuli and the central response boxes could require the rats to pay a significant amount of attention to the task and the severe performance deficits with the inactivations of the dorsal hippocampus could be mainly attributable to attention deficits. In order to test this hypothesis, we trained a separate group of rats (*n* = 6) in the cVCRS task in which the contextual stimulus was presented in the center touchscreen as a contextual background of the response boxes (Figure 1B *left*). We reasoned that presenting the contextual stimulus and the response boxes in the same screen should significantly reduce the attention load as compared to the pVCRS task.

**Figure 4:**
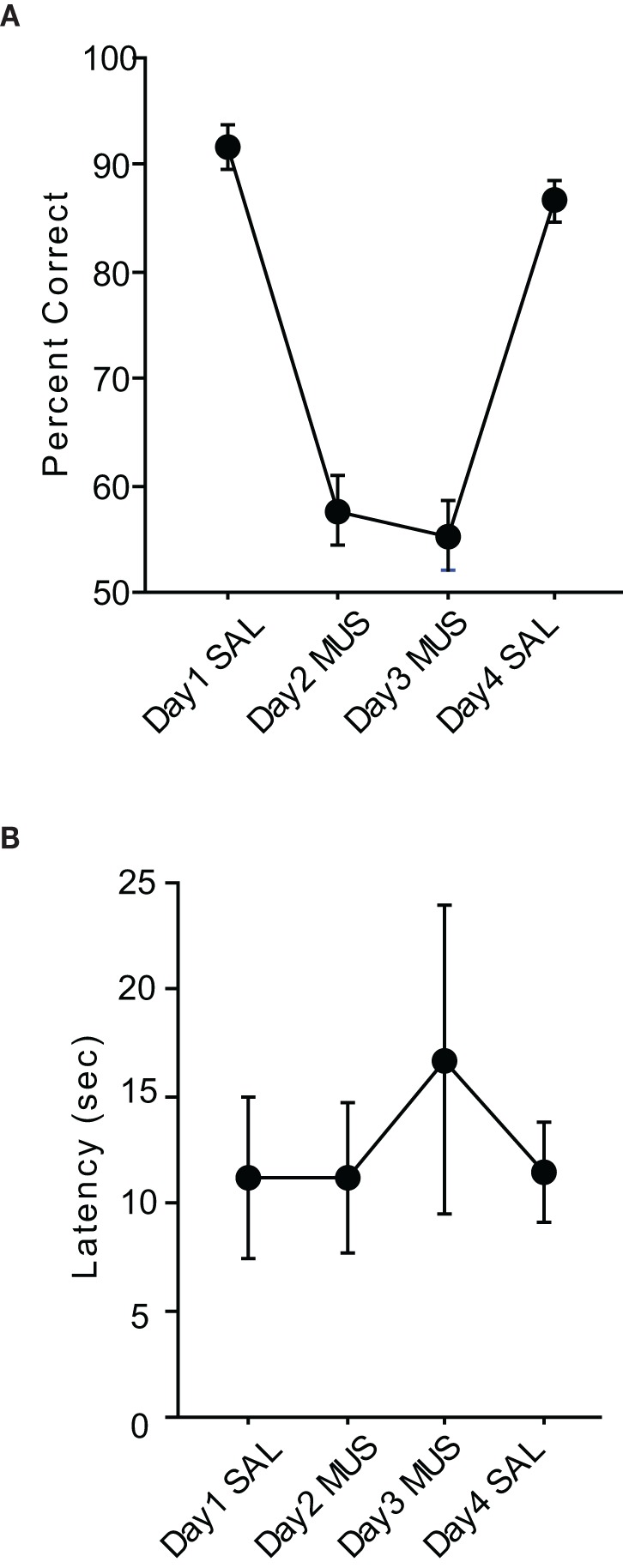
**Performance in the cVCRS task. (A)** Rats with MUS injections in the hippocampi were also impaired in the cVCRS task as severely as in the pVCRS task. **(B)** Latency measures were not significantly different between SAL and MUS conditions. All graphs show mean ± s.e.m.

The rats showed approximately 90% correct performances with SAL injections on average (Figure 4A). However, when MUS was injected for two consecutive days the same rats were impaired in performance as severely as in the pVCRS task. When SAL was injected on the last day of the injection schedule, the performance recovered to the previous SAL condition levels. The repeated measures of ANOVA showed that the drug condition had a significant effect [*F*_(3, 15)_ =54.66, *p* < 0.0001]. *Post-hoc* comparisons (Tukey-Kramer) showed all SAL injection showed significant differences with all MUS injections (*p* < 0.01). However, there was no significant difference between pre- and post-MUS injections. Also there was no significant difference on the first day and the last day of SAL injections as well. To see whether there were generic sensorimotor deficits when MUS was injected, the response latencies during SAL and MUS injections were compared. There were no significant differences between drug conditions [*F*_(3, 15)_ = 1.02, *p* = 0.41; Figure 4B]. We also calculated the response bias by taking the absolute difference between the numbers of left and right choices divided by the total trials. So if the rat chose only one side during the task the bias would be one and if it chose both sides evenly, the bias would be zero. The bias in SAL conditions stayed below 0.15 but the MUS conditions stayed over 0.5. In a repeated-measures ANOVA, there was a significant effect of drug condition [*F*_(3, 15)_ = 12.64, *p* < 0.001]. *Post-hoc* comparisons (Tukey-Kramer) showed significant differences between all SAL and MUS conditions (*p*-values < 0.05). However, there was no significant difference between first day of SAL and the last day of SAL. Also the pre- and post-condition of MUS had no significant difference.

The severe deficits in performance in the cVCRS task were observed similarly when the white rectangular response boxes were absent in the task (Figure 1B *right*). Specifically, we tested whether the poor performance with the MUS injection was attributable to the impairment in disambiguating the paired associations between the visual contexts and the identical visual objects (i.e., response boxes) since the hippocampus is also important for contextual disambiguation of objects (Lee and Solivan, 2008, 2010; Kim et al., 2011). This was tested by removing the response boxes in the cVCRS task in a separate group of rats. The rats with MUS infusions in the hippocampi in the no-response-box version of the cVCRS task were impaired as severely as in the original cVCRS task [*F*_(3, 9)_ = 12.21, *p* = 0.001, repeated-measures ANOVA]. This implies that the context-associated behavioral selection itself was impaired in the hippocampal-inactivated rats and the deficits were not due to ambiguities that might have caused by discriminating identical objects for making discrete responses.

Overall, the results from the cVCRS task strongly suggest that attention deficit was unlikely to explain the results of the pVCRS task because the hippocampus was essential for making behavioral choices using visual contextual stimuli regardless of whether the stimuli were presented peripherally or centrally.

### The hippocampal roles for perceptually discriminating visual contexts was minimal

The rats with MUS injections in the hippocampus might have been impaired in performance for the pVCRS and cVCRS tasks because they were unable to perceptually discriminate the two visual patterns under MUS inactivations of the hippocampus. This was tested in the VPD task (Figure 1C) in which the two visual contextual stimuli used in the VCRS tasks were presented simultaneously via the peripheral monitors. The rats were simply required to turn to the side where the rewarding visual stimulus was presented once reaching the choice point of the T-track. In contrast to the VCRS tasks in which contextual stimuli were used as a conditional cue for guiding the response selection behavior, only a perceptual discrimination of the visual stimuli was required in the VPD task.

**Figure 5:**
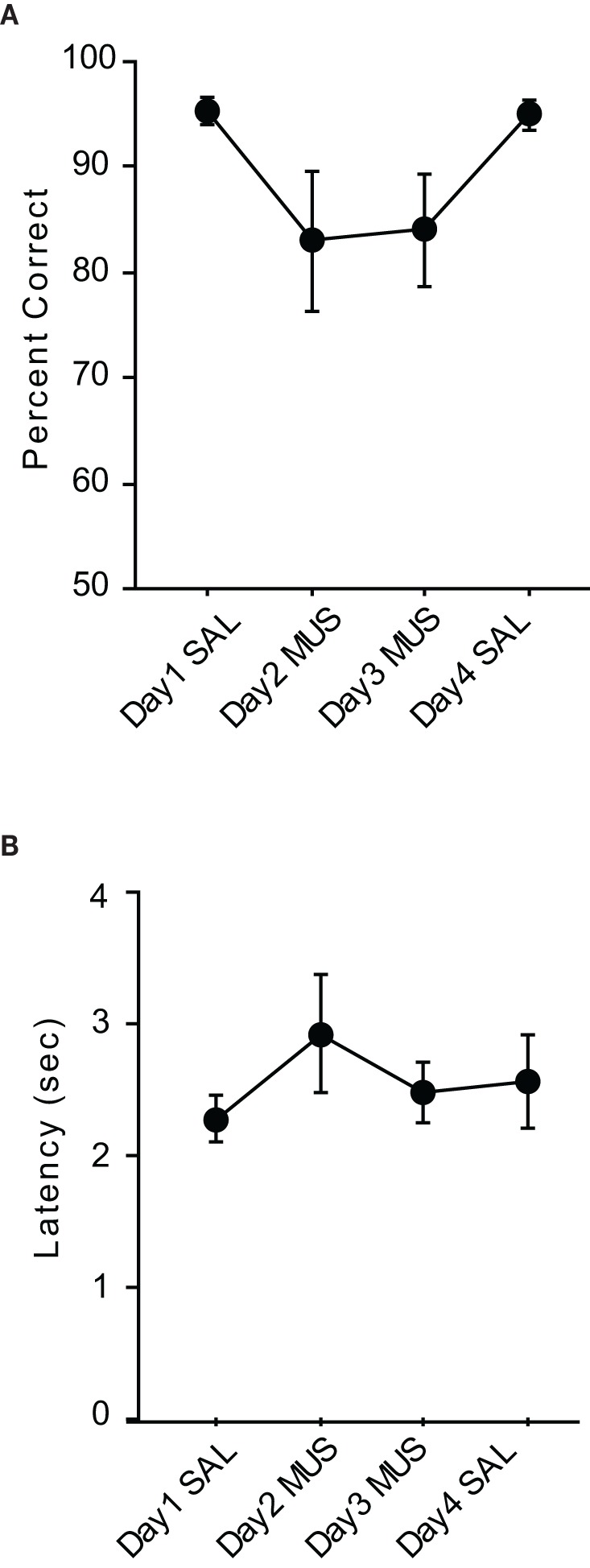
**Performance in the VPD task. (A)** Rats with SAL injections in the hippocampi showed almost perfect performances with little variances and also showed robust performances (approximately 85% correct) with MUS injections compared to the chance-level performances in the VCRS tasks with MUS infusions. **(B)** Latency measures were not different between SAL and MUS conditions. All graphs show mean ± s.e.m.

**Figure 6:**
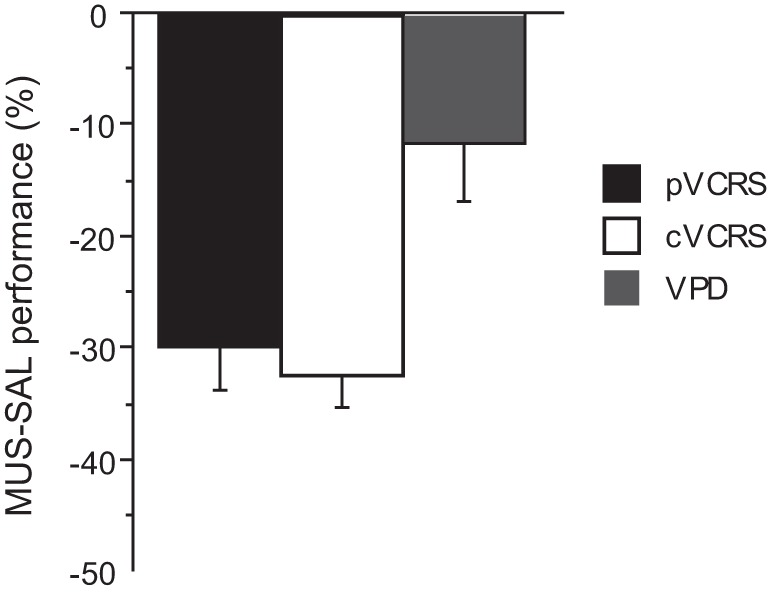
**Comparison of the performance drops with MUS-compared to SAL-injected conditions across the tasks.** The ordinate represents the difference in average performance (% correct) between the drug conditions on each task. Note the bigger drops in performance with MUS in the VCRS tasks as compared to the VPD task. All graphs show mean ± s.e.m.

The average performance of the rats during the injection schedule all stayed above 80% in the VPD task (Figure 5A). Since the rats showed over 95% average performance with minimal variance when SAL was injected, there was a significant effect of drug condition [*F*_(3, 15)_ = 3.59, *p* < 0.05]. Despite the mild performance deficits observed with MUS injections in the VPD task, however, the average decline in performance between SAL and MUS conditions was approximately only 10% in the VPD task, whereas approximately 30% decline in performance was observed in the MUS conditions in the VCRS tasks (Figure 6). An ANOVA showed a significant effect of task [*F*_(2, 15)_ = 7.85, *p* < 0.01] and a subsequent *post-hoc* test (Tukey-Kramer) revealed a significant difference in the performance decline measure between the VPD task and each of the VCRS tasks (pVCRS, cVCRS; *p*-values < 0.01). No significant difference was found between the two pVCRS and cVCRS tasks. The response latencies were not significantly different between SAL and MUS conditions (Figure 5B). The results overall suggest that the hippocampus was not as significantly required as in the VCRS tasks for the perceptual discrimination of the visual contexts.

## Discussion

In the current study, we tested whether the hippocampus was required for making discrete behavioral choices in association with the visual contextual stimuli presented in the background. Our new testing paradigm brings significant advantages including the precise control of onsets and offsets of stimuli and the parametric manipulations of stimulus features. Most of all, an experimenter can gain more confidence in the relationships between what animals actually see and what those animals use as a critical cue in the task as opposed to other prior contextual studies in which such relationships were vaguely defined. The animals with MUS-inactivations in the dorsal hippocampi exhibited severe performance deficits in the VCRS tasks. The performance was impaired regardless of whether the contextual stimuli were presented centrally or peripherally in relation to the response selection area as long as the visual contexts were presented in the background of the behavioral selection area. Inactivating the hippocampus, however, caused only mild deficits in the VPD task in which the need for response selection was minimal because the two different visual stimuli were pitted against each other on each trial and the rats were required to simply approach one of the visual stimuli associated with the reward. The results from our study suggest that the hippocampal functions can be tested powerfully by using pure visual stimuli as in primate and human studies and revealed some important properties regarding the contextual information processing in the hippocampus.

With respect to the source of deficits in the VCRS task, the hippocampal-inactivated rats could be perceptually impaired in discriminating visual stimuli presented in the LCD screens. However, the robust performance of the MUS-infused rats in the VPD task and the literature (Prusky et al., 2004; Forwood et al., 2005; Lee and Solivan, 2008; Talpos et al., 2009) make this generic perceptual account unlikely. On the other hand, one may need to take caution at the same time in directly comparing the results from the VPD task and VCRS task because there were some notable differences between the two tasks. The most important difference would be that all stimuli (two visual contexts) and all possible responses (two arms of the T-track) were available on each trial in the VPD task, whereas only a single visual context cued one of the response candidates (response boxes) in a given trial in the VCRS task. In the VPD task, therefore, a successful perceptual discrimination between the two contexts naturally led the rat to the correct response, whereas this was not the case in the VCRS task. In the VCRS task, the rat must recognize the visual context successfully first and then should choose a proper response between the possible choices available. That is, “contextual cueing” of response was required in the VCRS task, but not necessarily in the VPD task. We observed severe deficits in performance upon MUS inactivations in the hippocampus only when the contextual cueing was required in the current study (regardless of whether the cueing was made peripherally or centrally).

Our VPD task was similar to the visual discrimination task conducted by the Prusky group (Prusky et al., 2004) in that two visual stimuli and two response candidates (adjacent alleys in a modified water maze) were available on every trial in the Prusky et al. study as in the VPD task here. Specifically, black-and-white pictures composed of basic shapes were shown through 17-inch monitors in that study and the rats performed a delayed match-to-sample (MTS) task in a modified water maze and hippocampal-lesioned rats showed delay-dependent impairment in performance. The hippocampal-lesioned rats in the Prusky group's study showed robust performance (~80% correct) and this level of performance is similar to what we observed in the VPD task. In the Prusky et al. study, the performance dropped to the 60% level only when delays longer than 1 min were imposed between sample and test phases. The results further support our conjecture that the hippocampus is not required when simple perceptual discriminations are required for visual contextual stimuli (unless significant delay is imposed before response selection).

Hippocampal functions in learning and memory have been studied routinely using spatial navigation paradigms in rodents. Many different mazes were used for testing the navigational capabilities of rodents (mostly rats). Why is the hippocampus necessary for spatial navigation? What information processing in the hippocampus makes the region essential for navigation? We propose that the hippocampus is critically needed during spatial navigation mainly because a series of critical response selections (e.g., left turn *versus* right turn) need to be made upon encountering visual contexts at critical junctures in the course of wayfinding. It is well known that, when available, rats depend more on visual contextual cues in the environment for a successful navigation than on other cues such as idiothetic or local cues (Olton and Samuelson, 1976; Suzuki et al., 1980; Maaswinkel and Whishaw, 1999). In the central platform of a radial 8-arm maze, for example, the rat must choose a proper response between adjacent arms in a conditional manner considering the allocentric visual contextual cues in the environment. We would argue that our VCRS task provides a more controlled way of testing the critical cognitive processes required during spatial navigation. Specifically, the contextual stimuli presented in the LCD screens in the VCRS task may be equivalent to particular views of allocentric visual cues from the central platform in a radial-arm maze. Making a behavioral selection between the left and right response boxes in the VCRS task then is similar to choosing between adjacent arms in the radial maze. Similar cognitive processes can be identified in almost all types of mazes that require spatial navigations. Only two visual contexts were used in the current study but we know that rats can learn discrete conditional responses for multiple visual contexts in the current VCRS task (unpublished observations). Testing the rat's capabilities for making discrete left and right choices that are contingent upon visual contexts across trials may well be equated with the animal making correct turns successively at critical junctions in a real maze. On the other hand, the VPD task can be considered similar to a visually guided navigation (“cue-navigation”) in a Morris water maze task (Morris et al., 1982). It is well known that the hippocampus is not needed for this type of navigation and our results from the VPD task also match the literature. In comparison to using a real maze, however, the biggest advantage of using computer-controlled visual stimuli as contextual cues as in the current study is that the stimulus (visual context) that determines the behavioral selection is clearly defined in our task.

The VCRS task in the current study can be a valuable tool for studying dynamic contextual information processing in the hippocampus especially when it is combined with electrophysiological recording techniques in freely moving animals. For example, when a neuron fires selectively in one context (but in a relatively reduced fashion in another context) in a traditional hippocampal recording paradigms (Mizumori et al., 1999; Hayman et al., 2003; Moita et al., 2003; Leutgeb et al., 2004), it is difficult to pinpoint which contextual features of the environment make the neuron fire specifically in a certain context. In contrast, since the background visual context was the only information that can be used to solve the problem for each trial in the VCRS task, more straightforward relationships between neural activity and contextual representation can be studied. With an additional advantage of being able to present contextual stimuli that are more parametrically and quantitatively manipulated (for example, to construct various levels of ambiguous versions of the stimuli) on a trial-by-trial basis, the current behavioral paradigm can diagnose the behavior of a network more flexibly.

### Conflict of interest statement

The authors declare that the research was conducted in the absence of any commercial or financial relationships that could be construed as a potential conflict of interest.
